# French low-input winegrowing demonstration farms: A dataset of their operations traceability and sustainability performances

**DOI:** 10.1016/j.dib.2024.110415

**Published:** 2024-04-15

**Authors:** Olivier Nefti, Nicolas Chartier, Xavier Reboud, Thibault Peyrard, Laurent Deliere

**Affiliations:** a SAVE, INRAE, Bordeaux Sciences Agro, ISVV, F-33140, 71 rue Edouard Bourleaux, 33140 Villenave d'Ornon, France; b Institut de l'Elevage-Agrapole, 23 rue Jean Baldassini, F-69364 Lyon Cedex 7, France; c AGROECOLOGIE, INRAE, DPA3P, 17 rue de Sully, 21065 Dijon, France

**Keywords:** Agroecology, Viticulture, Pesticide, Performance indicator, Farm input, Farming system, Integrated pest management

## Abstract

This article presents data on farming operations traceability and associated performances, for winegrowing systems with low phytosanitary inputs. 343 farms were sampled from the DEPHY network: a governmental initiative to produce references on phytosanitary-efficient cropping systems under real conditions of production. Data were collected every campaign between 2017 and 2020, by multiple extensionists who provide support to the voluntarily enlisted growers, in exchange for traceability of their practices and their commitment to reducing pesticide use. The dataset includes raw data of farming operations (date, machinery, inputs, products and doses, etc.), and performance indicators computed at farm level (Treatment Frequency Index, workload, expenses, greenhouse gas emissions, etc.). This information could be useful to researchers, policymakers and agricultural consultants. It provides leads to understand how winegrowers manage to successfully reduce their pesticide consumption, as well as assessing the triggers and entailments of such transitions.

Specifications Table

**Table:** 

Subject	Agronomy and Crop Science
Specific subject area	Sustainability performances and technical management routes of French wine-growing systems, voluntarily committed to reducing their phytosanitary inputs.
Data format	Raw, Analysed, Filtered
Type of data	Table
Data collection	Extensionists from the DEPHY network record annual farm management data on the “Agrosyst” information system (presented below), for each of the ten to twelve winegrowers they are consulting. Data is then computed into indicators by both the software, and agronomists using reference frames from collaborating institutions (mostly non-releasable). Published data are anonymised, and have been filtered to match a definite period: the 2017, 2018, 2019, and 2020 campaigns; together with an initial point (traceability for at least one campaign prior to the network integration). Variables were consolidated (selection, methodological adjustments, standardisation) and translated into English by the authors.
Data source location	Described systems were part of a DEPHY collective between 2015 and 2020, at least. The collectives are scattered across mainland France's 9 main winegrowing regions: Alsace-Lorraine, Bordeaux-Bergerac, Bourgogne-Jura-Savoie, Champagne, Charentes, Languedoc-Roussillon, Rhône-Provence, Sud-Ouest, and Val de Loire. Data is stored online, in the “Agrosyst” information system, property of INRAE (National Research Institute for Agriculture and Environment).
Data accessibility	Repository name: Recherche Data Gouv (DATA INRAE dataverse). Data identification number: 10.57745/2HITDV Direct URL to data: https://doi.org/10.57745/2HITDV

## 1 Value of the Data

• The database is a valuable source of information on wine-growing systems that are committed, within a network, to reducing their use of plant protection products.
• Such data is useful because it depicts agronomic and techno-economic performances of these systems, paired with detailed information on their farming practices through distinct years and agro-climatic contexts.
• It can be used by agronomists, and the scientific community to understand how winegrowers manage to reduce their use of pesticides, and to assess the impact of these changes through univariate and multivariate analyses.
• This dataset could be used to complete existing databases, or be improved upon by implementing new indicators and external references sources.

## 2 Background

This article describes data collected annually by the French DEPHY network (the DEPHY acronym standing for: Demonstrate, Experiment and produce references on Phytosanitary-efficient cropping systems). This network is a governmental initiative, launched in 2010 as part of a national plan to reduce pesticide use (ECOPHYTO). It consists of 3000 farms, gathered in 244 collectives across the country (in 2020). Farmers are voluntarily engaged in these collectives, and commit to evaluate self-chosen practices in order to reduce their pesticide use. They receive both collective and individual guidance by an extensionist, who also records exhaustive information on their farm management [1].

## 3 Data Description

**Fig. 1:**
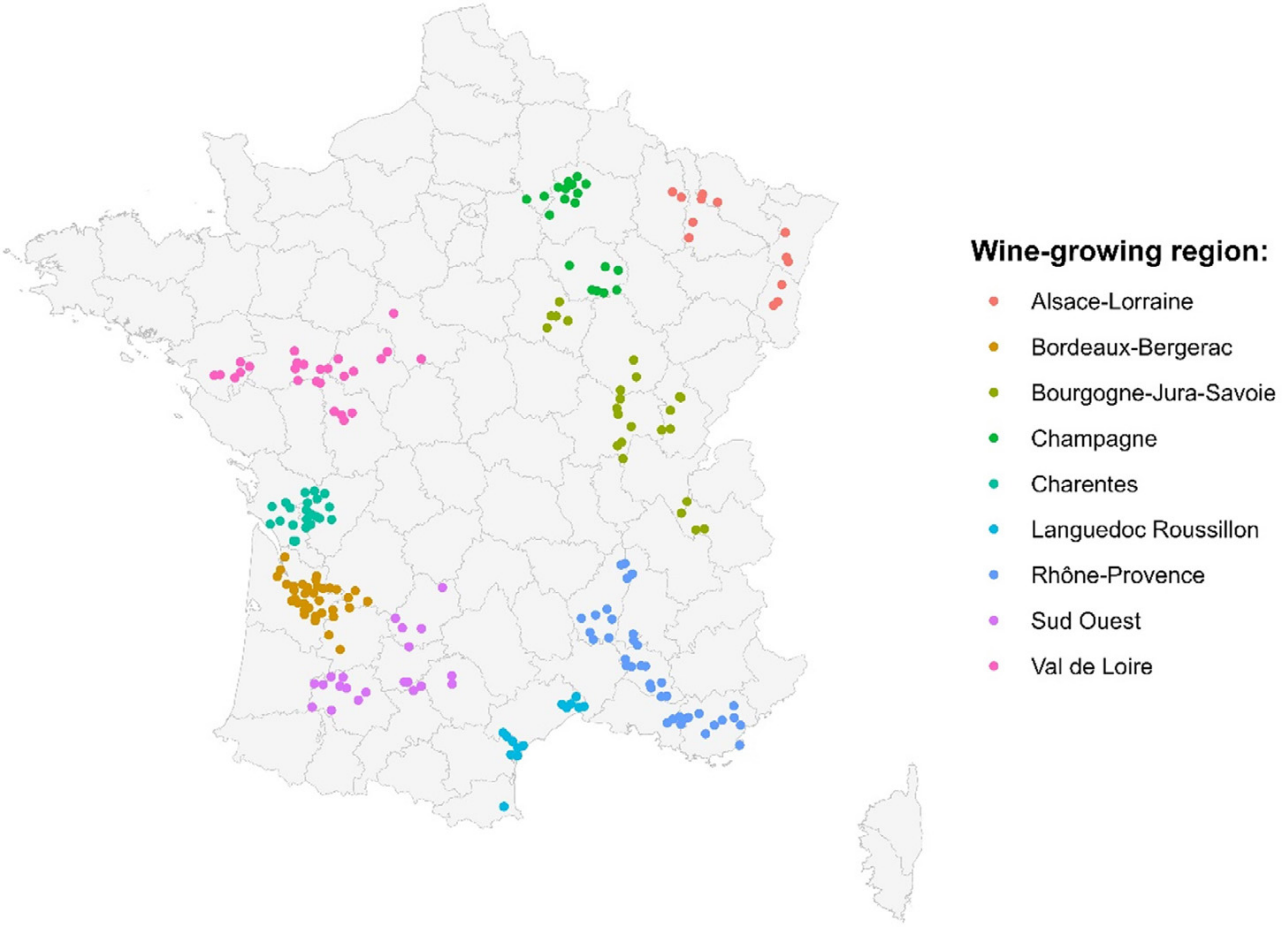
Location of the 343 sampled farms, and their winegrowing region.

Data presented here covers 343 of these farms, exclusively for the wine-growing sector, and campaigns from 2017 to 2020. They are scattered in every major winegrowing region of mainland France (Fig. 1).

Our dataset [2] encompasses three tables (.xlsx), and is structured as follows:

One table named Farm_operations_raw.xlsx; holding a record of cultural interventions performed on the studied farms. It consists of filtered data, from the extensionists’ raw inputs in the information system. In this spreadsheet, one line describes one input and/or one farming intervention for a single farm and campaign. Cultural interventions with multiple inputs are therefore displayed on as many lines, and are grouped by a common “*intervention_id*”. Most of the information provided here directly originates from software extractions and was nearly unprocessed. It includes contextual details such as region, grape variety, or production system (organic or conventional), as well as specifications about operations: dates, areas, machinery used, inputs, doses etc.

A second table: Farm_performances_aggregated.xlsx provides compiled scores for various performance indicators on each campaign of the sampled farms. Hence, one line describes one farm and one campaign. Indicators cover pesticide consumption (Treatment Frequency Index, Quantity of Active Ingredients, etc.); socio-technical parameters (workload, expenses, etc.); and environmental metrics (fuel consumption, greenhouse gas emissions). They result from the aggregation at farm level of the Farm_operations_raw.xlsx data, matched with reference sources (partially undisclosed).

A third table: Variables_description.xlsx, offers a concise glossary for every column label of the two previous files. When suitable, it also states the variables’ values and units, as well as the reference sources involved in the computation.

Both Farm_operations_raw.xlsx and Farm_performances_aggregated.xlsx are designed to allow a comparison between farm data, before and after the network monitoring. To that end, they include an “Initial point” for every wine-growing system, as a complement to the four studied campaigns. These initial points were recorded by extensionists in similar fashion and offer the same farm description data, yet one to three years prior to the network admission (cf. materials and methods).

## 4 Experimental Design, Materials and Methods

### 4.1 General context of raw data acquisition

#### 4.1.1 Functioning of the network, and data collection

DEPHY network consists of peer collectives, composed of a dozen farmers from a set area and agricultural sector. They are monitored by an extensionist leading the group through steady workshops (meetings, field trips, educational visits, etc.), and technical assistance on alternative practices. These “network engineers” are employed by independent organisations, and allocate half of their working time to group animation and data recording. For farmers, network membership is free but does not grant subsidy nor economic incentives. In return, they commit to partake in the collective project and test pesticide-efficient practices for a 5 years’ period. When a farm joins in a collective, the DEPHY extensionist performs a diagnosis of its current practices (called “initial point”), and helps setting up a plan to reduce its pesticide use. Farmers can nevertheless operate freely, without any obligation to adhere strictly to this scheme. And while they are not required to commit their entire farm land to the network project, they must at least devote a homogeneous set of plots to it, called here the “wine-growing system”, which should account for a significant part of their farming activity [1]. In 2020, the supervised surface area represented on average 92 % of a farm's vineyard acreage, and 77 % of its total agricultural area.

From then on, management information is recorded yearly for these plots: characteristics, crop rotation and varieties, fleet of equipment, traceability of cultural operations, pest pressures, protection strategies, etc. All data are entered online by the extensionist, into the “Agrosyst” information system, designed for this purpose by INRAE: France's national research institute for agriculture and environment [3].

#### 4.1.2 Farm initial point and general data entry instructions

In order to describe how a wine-growing system was managed before it entered the network, engineers proceed in one of two ways, depending on the information available to them. Their first option is to describe one (and only) fictitious traceability of interventions, corresponding to a “**summarised**” representation of the monitored plots during previous campaigns (1 to 3). When more precise data is available, the second option is to enter the actual traceability of equipment and operations for as much campaign prior to network entry as possible (also with a maximum of 3). In the presented dataset, farm initial points described as such are identified as “**averaged**”, since their compiled performance indicators refer to one-year average values for the specified period (cf. the “Computation of farm performances'' paragraph of this section). Most sampled farms joined the network either in 2012 or in 2016; hence, the majority of initial points cover the 2009, 2010, 2011 and 2013, 2014, 2015 periods.

Once the initial point is assessed, extensionists carry on with the farm description, as they are required to annually update information in Agrosyst. Most data are filled in via drop-down menus, drawing upon information supplied by a range of reference frameworks. These reference sources are provided by partners of the DEPHY network, for instance this dataset's variables involve the following databases:

- Baseline data on operations costs and agricultural mutual aid tariff scale, for all machinery equipment and their characteristics [4].
- Several annual studies by the company “TerreEtude,” providing yearly estimates of average costs for each plant protection input authorised for use in France [5].
- The PlantGrape inventory of French cultivated grape varieties [6], together with the national catalogue of approved varieties (GEVES) [7].
- The national repository of plant protection products, fertilisers and crop supports, adjuvants, mixed products and blends [8]

#### 4.1.3 Global data processing to assemble the dataset

This dataset [2] solely encompasses farms specialised in winegrowing, which had been filtered for the quality of their traceability data. The sampling consisted of manually ruling out farms that had joined or left the network between 2017 and 2020. Those for which traceability was incomplete or missing, for one of these campaigns or for the initial point, were also excluded. Data completeness was evaluated through the presence of key cultivation practices: such as pruning, weed management, phytosanitary treatments, etc. Thanks to this sampling process, out of 435 eligible farms, 343 were selected to establish the database. Two successive extractions have been made from the Agrosyst software to assemble the dataset: on the 30/06/2022 and 31/03/2023. After data curation, all tables have been translated to English, partially using the glossary of European “IPM works” project [9].

Traceabilities of farm operations were tallied in Farm_operations_raw.xlsx, for every sampled farms and period (Initial point + 2017, 2018, 2019, 2020). Displayed operations encompass all vineyard management interventions from pruning to harvest. Some practices were however omitted, as they were either uncommon (irrigation), or generic but not always specified by extensionists with sufficient consistency (trellis maintenance, re-planting, etc.). Harvest yields were not included in the table, as the wide range of units and maximum authorised yields induced by origin designations did not allow for sound data curation.

### 4.2 Computation of farm performances

In Farm_performances_aggregated.xlsx, most variables are derived from raw data, matched with several reference sources and aggregated at farm level. Some of them can be recalculated directly from Farm_operations_raw.xlsx.

#### 4.2.1 Pesticide consumption metrics

Treatment Frequency Index is the main indicator used in France, and in the DEPHY network to quantify pesticide use. It is rather focused on consumption and purpose than on environmental or health hazards, and can be interpreted as “the number of full-dose treatments per unit area during a crop season” [10]. For this dataset, it was calculated at farm level following Eq. (1). Here, adjuvants were not considered as actual plant protection products, and were therefore omitted from the computations.

(1) $$\mathrm{TFI}=\sum_{p}\left(\frac{\text{sprayed}\_\text{dos}e_{p}}{\text{approved}\_\text{dos}e_{p}}\right)\times \left(\frac{\text{sprayed}\_\text{are}a_{p}}{\text{total}\_\text{area}}\right)$$

The “approved_dose” is the maximal dose legally allowed to be applied per treatment, for a given product (p) and purpose. It is determined and regularly renewed by the National Agency for Social Security (ANSES), and stored in the EPHY ANSES database [8]. No matter the treatment date, each of this dataset's phytosanitary inputs were matched with the approved dose prevailing in 2022, using the *“PPP_MA_code”* identifier from Farm_operations_raw.xlsx. All figures being expressed per hectare, *“total_area”* was considered equal to 1, and the *“sprayed_area”* of a treatment was calculated via the intervention (i) and product (p) data of Farm_operations_raw.xlsx, following Eq. (2).

(2) $$\begin{array}{ccc} \mathrm{sprayed}\_\mathrm{are}a_{p} & = & \;\text{variety}\_\text{surface}\_\text{shar}e_{i}\times \text{spatial}\_\text{frequenc}y_{i}\times \text{temporal}\_\text{frequenc}y_{i}\\ & & \times \;\text{treated}\_\text{are}a_{p} \end{array}$$

TFIs provided in the table are only partial, since they were calculated for specific types of products, as detailed in Table 1:

**Table 1:** Partial TFIs displayed, and the products considered for their calculation.

Partial TFI	Plant Protection Products considered for calculation
TFI_be	All PPP, excluding those for which *“PPP_classification”* = “Biocontrol”
TFI_biocontrol	All PPP for which *“PPP_classification”* = “Biocontrol”
TFI_h_be	PPP with “PPP_type” = “HERBICIDAL”; and *“PPP_classification”* ≠ “Biocontrol”
TFI_f_be	“PPP_type” = “FUNGICIDAL” or “NATURAL_CONTROL_SYSTEM_STIMULATOR”; and *“PPP_classification”* ≠ “Biocontrol”
TFI_i_be	“PPP_type” = “INSECTICIDAL”; and *“PPP_classification”* ≠ “Biocontrol”

“Biocontrol” here refers to all plant protection products listed in the French ministerial register entitled as such, in 2022 [11]. This register features “macroorganisms, microorganisms, natural substances, chemical mediators and defence elicitors”. In addition, the register rules out substances that entail major externalities regarding human health or the environment. Hence, copper-based fungicides are not counted as “biocontrol”, but sulphur-based ones are. In the *“PPP_classification”* variable, they are opposed to Carcinogenic, Mutagenic and Reprotoxic products (CMR), which hold at least one of these hazards statements: H341, H350, H360, H360D, H360Df, H360FD, H361, H361d, H361f, H361fd, H362 [12].

Total quantities of active ingredients (QAI) track the total mass of biocide molecules applied per hectare during one season, including adjuvants. They also result from the aggregation of all products (p) applied during a campaign (Eq. (3)). Formulations of products and concentrations were extracted from EPHY ANSES database [8]. Again, partial values are provided by computing only products that contain noteworthy substances: such as copper (*“copper_q_PPP”*), sulphur (*“sulfur_q_PPP”*), glyphosate (*“QAI_glypho”*) and CMR classified molecules (*“QAI_CMR”*).

(3) $$\mathrm{QAI}=\;\sum_{p}Dose_{p}\times concentration_{p}\times sprayed\_area_{p}$$

A similar methodology, although based on user entries instead of reference frames, was employed to report copper and sulphur quantities brought in by fertilisers: *“copper_q_fertilisers”* & *“sulfur_q_fertilisers”*.

#### 4.2.2 Workforce requirement indicators

Indicators pertaining mechanized operations vastly draw on external data from the operation costs reference frame [4]. When an extensionist fills in the details of farms’ equipment, the software automatically determines standard characteristics for each of its agricultural machinery, based on this external source. For instance, the “work_rate” (h/ha) of mechanical interventions (i) used in equation 4 originates from this reference frame, it is therefore not included in our data tables. Values for “reference_fuel_price” from Eq. (9); “engine_power”, “engine_load” and “reference_fuel_consumption” (Eq. (10)) are not featured either, for the same reasons. This also applies to the “purchase_price” and “amortisation_rate” (Eq. (12)), as well as the “repair”, “tyre” and “engine_oil” expenses (Eq. (13)).

Additionally, all labour related indicators are expressed off-harvest (Eqs. (4)–(6),(8)), since the work-rates of manual and mechanical harvesting are very uneven, and cannot be compared. The “labour_unit” variable (Eq. (6)) depicts a theoretical number of full-time employees required to operate one hectare during one campaign, using 1600 h as the annual working time reference in France [13].

(4) $$\begin{array}{ccc} \mathrm{Equipment}\_\mathrm{operating}\_\mathrm{time} & = & \sum_{i}\text{variety}\_\text{surface}\_\text{shar}e_{i}\times \text{spatial}\_\text{frequenc}y_{i}\\ & & \times \;\text{temporal}\_\text{frequenc}y_{i}\times \text{work}\_\text{rat}e_{i} \end{array}$$

(5) $$\begin{array}{ccc} \mathrm{Manual}\_\mathrm{labour}\_\mathrm{time} & = & \sum_{i}\text{variety}\_\text{surface}\_\text{shar}e_{i}\times \text{spatial}\_\text{frequenc}y_{i}\\ & & \times \;\text{temporal}\_\text{frequenc}y_{i}\times \text{nb}\_\text{worker}s_{i}\times \text{work}\_\text{rat}e_{i} \end{array}$$

(6) $$\mathrm{Labour}\_\mathrm{unit}=\frac{\text{Equipment}\_\text{operating}\_\text{time}+\text{Manual}\_\text{labour}\_\text{time}}{1600}$$

#### 4.2.3 Production costs

Purchase expenses for all plant protection products are calculated thanks to standard reference prices, set for each year over the whole country. These fiducial values are provided by the “TerreEtude” market research firm [5], and could not be disclosed with the dataset. This cost item only applies to the products (p) actually used during the campaign (adjuvants included), the aggregation at farm level was then processed as in Eq. (7).

(7) $$\mathrm{PPP}\_\mathrm{expenses}=\sum_{p}dose_{p}\times price_{p}\times \text{sprayed}\_\text{are}a_{p}$$

Mechanisation costs are interpreted as the sum of outlays for running and maintaining a farm's fleet of equipment. They partly derive from equipment operating time and fuel consumption (Eqs. (4) & (10)) of interventions (i), but also from characteristics and standard costs of equipment (e), as detailed in Eqs. (8)–(13).

(8) $$\mathrm{Mechanisation}\_\mathrm{cost}s_{i}=\sum_{i}\text{fuel}\_\text{cos}t_{i}+\text{intervention}\_\text{are}a_{i}\times \left[\text{fixed}\_\text{cost}s_{i}+\text{variable}\_\text{cost}s_{i}\right]$$

With

(9) $$\mathrm{fuel}\_\cos t_{i}=fuel\_consumption_{i}\times \text{reference}\_\text{fuel}\_\text{price}$$

(10) $$\begin{array}{ccc} \mathrm{Fuel}\_\mathrm{consumption} & = & \sum_{i}\text{equipment}\_\text{operating}\_\text{tim}e_{i}\times \text{engine}\_\text{powe}r_{i}\;\times \text{engine}\_\text{loa}d_{i}\\ & & \times \;\text{reference}\_\text{fuel}\_\text{consumption} \end{array}$$

(11) $$\mathrm{intervention}\_\mathrm{are}a_{i}=\;\left[\text{variety}\_\text{surface}\_\text{shar}e_{i}\times \text{spatial}\_\text{frequenc}y_{i}\times \text{temporal}\_\text{frequenc}y_{i}\right]$$

(12) $$\mathrm{fixed}\_\mathrm{cost}s_{i}=\sum_{e}\frac{\text{purchase}\_\text{pric}e_{e}\;\times \text{amortisation}\_\text{rat}e_{e}}{\text{annual}\_\text{utilisatio}n_{e}}$$

(13) $$\mathrm{variable}\_\mathrm{cost}s_{i}=\sum_{e}\text{repair}\_\text{expense}s_{e}+\;\text{tyre}\_\text{expense}s_{e}+\;\text{engine}\_\text{oil}\_\text{expense}s_{e}$$

Labour costs were estimated using calculated work times (Eqs. (4). and (5).) and standard hourly wage rates; namely 14€ per hour for manual operations, and 16€ per hour for mechanised ones.

#### 4.2.4 Environmental performances

Greenhouse gas emissions is the main metric used in the dataset to measure farms’ environmental externalities. They are calculated using fuel and pesticide consumption data, according to the “GEST'IM” life cycle assessment method [14]. Reference values from this framework encompass global warming potential for CO_2_, CH_4_ and N_2_O emissions; and are expressed in kg CO_2_ equivalent per litre of fuel (Eqs. (13)–(15)).

Emissions induced by fuel consumption were computed off-harvest (HE standing for Harvest Excluded), and are presented through two indicators: *“direct_GHG_fuel_HE '' and “indirect_GHG_fuel_HE”*. The former describes on-farm discharges, through combustion of energy resources during mechanical operations, and the latter consists of the up-stream emissions that occurred during the production and transportation of the consumed fuel. For both of them, it was assumed that only “off-road diesel” was consumed in the sampled farms, rather than other fuel types (petrol, bio-ethanol, etc.).

(14) direct_GHG_fuel_HE=fuel_consumption×2.646

(15) indirect_GHG_fuel_HE=fuel_consumption×0.425

Contribution of pesticides to greenhouse effect is also provided, solely as up-stream emissions during industrial production. They are assessed thanks to average emission potential values per product type: fungicide, herbicide, insecticide, and “others” (see Eq. (16).).

(16) $$\mathrm{indirect}\_\mathrm{GHG}\_\mathrm{PPP}=\sum \left\{ \begin{array}{c} \text{QAI}\_\text{fungicide}\times 6.009\\ \text{QAI}\_\text{herbicide}\times 8.985\\ \text{QAI}\_\text{insecticide}\times 25.134\\ \text{QAI}\_\text{other}\times 8.478 \end{array}\right.$$

#### 4.2.5 Census data on grower's campaign evaluation

Lastly, qualitative data about growers’ **campaign evaluation** is provided in Farm_performances_aggregated.xlsx. In addition to the recording of intervention traceability, network engineers schedule individual meetings once a year to take stock of the past agricultural season for each of their growers. These meetings consist of formal exchanges, whose transcripts are also entered in the information system via drop-down menus. Some of this information have been integrated to the dataset [2], namely the following variables: *“yield_%_target”, “yield_loss_causes”, “downy_mildew_pressure”, “downy_milew_control”, “powdery_mildew_pressure”*, and *“powdery_milew_control”* (cf. Variables_description.xlsx for further description and values). Such information was not collected during the initial point assessment, and therefore unavailable for this period. For the 2017-2020 period, data is also missing for a small number of farms and certain campaigns (7 % overall) because it was not collected.

## Limitations

The main limitations of the dataset lie in the quality of data for “initial points”. It was acquired via two different methodologies (summarised and averaged), and often covers different periods from one farm to another. This information was included in the dataset in order to provide insights on how farming practices evolved in a given farm under network supervision. But unlike the 2017, 2018, 2019, 2020 campaigns; it may hence not be suitable for comparing farms with one another.

In Farm_performances_aggregated.xlsx, winegrowers’ campaign evaluation items also hold limitations, in terms of quality and quantity of data. They bring strictly declarative data; which could not be collected for every farm and period. When available, it however offers supplementary contextual information, and may help interpret some of the numerical values.

## Ethics Statement

The authors confirm they have read, and follow, the ethical requirements for publication in Data in Brief. The work published in this paper and dataset did not involve human subjects, animal experiments nor data collected from social media platforms.

## CRediT authorship contribution statement

**Olivier Nefti:** Conceptualization, Data curation, Writing – original draft. **Nicolas Chartier:** Conceptualization, Methodology, Resources, Writing – review & editing. **Xavier Reboud:** Conceptualization, Methodology, Resources, Software. **Thibault Peyrard:** Conceptualization, Methodology, Resources, Software, Writing – review & editing. **Laurent Deliere:** Conceptualization, Supervision, Writing – review & editing.

## Data Availability

DEPHY FERME network's low input winegrowing systems: performances and traceability (Original data) (Recherche Data Gouv - Data INRAe)
